# Eight Weeks of Supervised Pulmonary Rehabilitation Are Effective in Improving Resting Heart Rate and Heart Rate Recovery in Severe COVID-19 Patient Survivors of Mechanical Ventilation

**DOI:** 10.3390/medicina58040514

**Published:** 2022-04-05

**Authors:** María Fernanda del Valle, Jorge Valenzuela, Gabriel Nasri Marzuca-Nassr, Consuelo Cabrera-Inostroza, Mariano del Sol, Pablo A. Lizana, Máximo Escobar-Cabello, Rodrigo Muñoz-Cofre

**Affiliations:** 1 Servicio de Medicina Física y Rehabilitación, Hospital el Carmen, Maipú 9251521, Chile; fer.delvalle91@gmail.com (M.F.d.V.); jorge.valenzuelav@redsalud.gob.cl (J.V.); consuelocabrera@hotmail.cl (C.C.-I.); 2 Departamento de Medicina Interna, Facultad de Medicina, Universidad de La Frontera, Temuco 4781218, Chile; gabriel.marzuca@ufrontera.cl; 3 Centro de Excelencia en Estudios Morfológicos y Quirúrgicos, Universidad de La Frontera, Temuco 4811230, Chile; mariano.delsol@ufrontera.cl; 4 Laboratory of Epidemiology and Morphological Sciences, Instituto de Biología, Pontificia Universidad Católica de Valparaíso, Valparaíso 2373223, Chile; pablo.lizana@pucv.cl; 5 Laboratorio de Función Disfunción Ventilatoria, Departamento de Kinesiología, Universidad Católica del Maule, Talca 3480112, Chile; maxfescobar@gmail.com; 6 Posdoctorado en Ciencias Morfológicas, Universidad de La Frontera, Temuco 4811230, Chile

**Keywords:** heart rate, COVID-19, pulmonary rehabilitation

## Abstract

*Background and Objectives*: Patients who survive severe COVID-19 require significant pulmonary rehabilitation. Heart rate (HR) has been used as a safety variable in the evaluation of the results of interventions in patients undergoing pulmonary rehabilitation. The aim of this research was to analyse HR during a pulmonary rehabilitation program in post-severe COVID-19 patients who survived mechanical ventilation (MV). The study includes the initial and final evaluations and aerobic training sessions. *Materials and Methods:* Twenty patients (58 ± 13 years, 11 men) were trained for 8 weeks. A 6-minute walk test (6 MWT) was performed and, subsequently, a supervised and individualised training plan was created. Resting heart rate (RHR), heart rate recovery (HRR), heart rate at minute 6 (HR6 min) and the product of HR6 min and systolic blood pressure (HR6 min^x^SBP) were measured at 6 MWT. In addition, HR was measured at each training session. *Results:* After 8 weeks of pulmonary rehabilitation, patients decreased their RHR from 81.95 ± 9.36 to 73.60 ± 9.82 beats/min (*p* < 0.001) and significantly increased their HRR from 12.45 ± 10.22 to 20.55 ± 7.33 beats/min (*p* = 0.005). HR6 min presented a significant relationship with walking speed and walked distance after the pulmonary rehabilitation period (r = 0.555, *p* = 0.011 and r = 0.613, *p* = 0.011, respectively). HR6 min^x^SBP presented a significant relationship with walking speed and walked distance after training (r = 0.538, *p* = 0.014 and r = 0.568, *p* = 0.008, respectively). In the pulmonary rehabilitation sessions, a significant decrease in HR was observed at minutes 1, 6 and 15 (*p* < 0.05) between sessions 1 and 6 and at minute 1 between sessions 1 and 12. *Conclusions:* Eight weeks of individualised and supervised pulmonary rehabilitation were effective in improving RHR and HRR in COVID-19 patients surviving MV. HR is an easily accessible indicator that could help to monitor the evaluation and development of a pulmonary rehabilitation program in COVID-19 patients who survived MV.

## 1. Introduction

COVID-19 is a disease caused by the SARS-CoV-2 virus, belonging to the coronavirus family [1]. In December 2019, the World Health Organization (WHO) in China warned about patients with pneumonia of an unknown aetiology and the first case in Chile was registered on 3 March 2020 [1,2]. This marked the beginning of a pandemic that has since affected the entire world [3]. The projections of the functional consequences of this syndemic are still a matter of speculation [4].

Due to severe respiratory symptoms and, in some cases, acute respiratory distress, patients with COVID-19 may require prolonged mechanical ventilation (MV). In addition to MV being an invasive procedure, there are cases where its disconnection can take time. The consequences of prolonged connection to MV generate the need for pulmonary rehabilitation during and after hospitalization [5]. These MV-related complications can include respiratory problems, cognitive problems, myopathies, neuropathies, joint pain, muscle pain, physical deconditioning and cardiac disorders [4,5,6]. In this sense, the American Thoracic Society-European Respiratory Society (ATS-ERS) suggests that aerobic exercise should be part of a pulmonary rehabilitation program. However, this should be structured individually, after a formal evaluation, due to the cardiorespiratory sequelae caused by COVID-19 [7]. Therefore, heart rate (HR) control in the training evaluation and implementation process would be useful [8], considering the linear relationship between oxygen uptake and cardiac output or HR during functional tests [9].

Some symptoms of COVID-19 can last beyond the period of acute infection, with exercise intolerance standing out as the most frequent finding [7,8,10]. This can occur together with chest pain, dyspnoea, palpitations or even postural orthostatic tachycardia [7]. Exercise intolerance can cause a limitation of activities, resulting in an effort that goes beyond daily life or inactivity [7,8]. Here, the restriction of movement becomes a confounding factor instead of a protective factor [9]. Therefore, the monitoring of HR upon return to exercise is recommended, with the purpose of observing the exercise behaviour of patients who survived COVID-19 [8] and also to determine whether there is an indirect impact of pulmonary rehabilitation on the cardiopulmonary system.

Baseline fitness assessment through the 6-minute walk test (6 MWT) and aerobic training are part of most pulmonary rehabilitation programs [7,11]. Thus, changes in HR with various interventions that increase physical workload may be useful in assessing an exercise training risk profile [8,11]. It has been reported that the decrease in HR at rest related to physical activity decreases the incidence of cardiovascular diseases, in addition to having a positive impact on all-cause mortality [12,13]. On the other hand, heart rate recovery (HRR) shows autonomic activity in the cardiovascular system and has also been shown to be a predictor of morbidity and mortality in patients with heart failure [14]. In this context, the aim of this study was to analyse HR during a pulmonary rehabilitation program in post-severe COVID-19 patients who survived MV, including the initial and final evaluations and aerobic training sessions. In this sense, our research hypothesis is that the intervention, through an 8-week pulmonary rehabilitation program, would decrease resting heart rate (RHR) and improve HRR.

## 2. Materials and Methods

### 2.1. Participants

This was a prospective study, where the sample was for convenience and the recruitment of participants to the pulmonary rehabilitation program was in accordance with bronchopulmonary dysplasia. Twenty patients (58 ± 13 years old; 11 men and 9 women) diagnosed with COVID-19 were included in the present study. This research was approved by the Scientific Ethics Committee of the Central Metropolitan Health Service (Resolution No. 048975). In addition, all patients read and signed an informed consent agreement. The inclusion criteria were as follows: (a) diagnosis of severe/critical COVID-19, (b) requirement of MV with orotracheal intubation, (c) hospital discharge, (d) control with a cardiologist and normal electrocardiogram and (e) being in health control at the Carmen Hospital in Maipú, Santiago, Chile. Patients who did not understand commands were excluded. All participants underwent 8 weeks of individualised and supervised pulmonary rehabilitation. In these 8 weeks, 2 evaluation sessions (PRE and POST), 12 exercise sessions and 2 control sessions (after completion of pulmonary rehabilitation) were included. Before (PRE) and after (POST) pulmonary rehabilitation, the walked distance with the 6 MWT was evaluated, and the RHR, HRR, heart rate at minute 6 (HR6 min) and product of the HR6 min with systolic blood pressure (SBP) (HR6 min^x^SBP) were measured in this test. Ventilatory capacity through spirometry was also performed before and after 8 weeks of pulmonary rehabilitation. In addition, an incremental and continuous test was carried out to design the pulmonary rehabilitation sessions. HR was also measured at each training session.

### 2.2. Test Day

The test period consisted of two identical evaluation days before and after the pulmonary rehabilitation period. The participants arrived at the hospital and underwent an evaluation of their baseline parameters (blood pressure, HR, weight and height) and then performed a spirometry test. After a 10-minute rest, a 6 MWT was performed with the measurement of the parameters indicated above before and after the test. The 6 MWT was used to measure the walked distance by each participant and their walking speed, which allowed the incremental test to be dosed according to the conditions of each participant. The continuous test was used to evaluate the duration of the maximum speed obtained in the incremental test. The three tests (6 MWT, continuous test and incremental test) were performed on the same day, with 10 min of rest between each one and always in the same order.

#### 2.2.1. Spirometry

Spirometric measurements were standardised according to the standards of the American Thoracic Society [15], incorporating national suggestions in relation to care due to the COVID-19 pandemic [16]. The patient, in a seated position, placed the pneumotachograph in his/her mouth and forced expiration was requested based on total lung capacity. The values of forced vital capacity (FVC), which is the volume that has been exhaled at the end of the first second of forced expiration (FEV1), and their ratio (FEV1/FVC) were considered. For this, a Medgraphics spirometer (CPFS/D USB 2.02, MGC Diagnostics Corporation, Saint Paul, MN, USA) was used [17]. 

#### 2.2.2. Walked Distance and Heart Rate Parameters

In this study, 6 MWT was held in a 30-metre-long corridor, free of traffic. According to Muñoz et al., with constant stimulation, patients were instructed to walk as many metres as possible during the corresponding six minutes [18]. Dyspnoea and lower limb fatigue were categorised using the modified Borg scale [19]. Oximetry was measured at the beginning and end of the test by a pulse oximeter (Nonin 7500^®^, Nonin Medical, Minneapolis, MN, USA). The walked distance was recorded in metres. HR was recorded using the Polar^®^ system (Polar^®^ FT2, Kempele, Finland). An elastic belt (Polar T31 transmitter, Polar Electro, Kempele, Finland) was attached to the participant’s chest at the level of the lower third of the sternum. In the 6 MWT, HR was recorded at rest, during the 6 MWT and in the recovery period (one minute after completion of the 6 MWT) [18,20]. The HRs used were the following: (1) HRH, (2) heart rate at minute 6 (HR6 min) [9], (3) HRR = HR at minute 6 of the 6 MWT minus the HR at one minute after the completion of the 6 MWT [20] and (4) the product of HR6 min and systolic pressure at minute 6 of 6 MWT (HR6 min^×^SBP) [13]. In addition, HR was measured in each pulmonary rehabilitation session; at minutes 1, 3, 6, 10, 15, 20, 25 and 30; and in recovery (1 and 5 min).

#### 2.2.3. Incremental Test

The incremental test was performed on a treadmill (Spirit CT800 212089^®^, Jonesboro, AR, USA). The loads used in each stage of the incremental test were calculated from the average speed obtained in the 6 MWT, with the method described above [21]. Using a known distance and a stopwatch, the time it took for the patient to travel 30 m was measured. Gait speed was calculated using the following equation: speed = distance/time (m/sec). Subsequently, the conversion was made to km/h, a unit to be programmed in the treadmill. The maximum speed or 100% was the average obtained from each turn. The initial stage was using 45% speed on the 6 MWT and an incline of 1. The speed was in-creased by 15% and the incline level was also increased every 1 min. Once 100% of the speed calculated through the 6 MWT was reached and in the absence of test suspension criteria, the speed continued to increase by 15% every minute [22]. HR, dyspnoea and fatigue were assessed at each minute of the session. The test was stopped when the patient presented dyspnoea or fatigue ≥ 7 points, a pulse saturometry of <91% and/or exceeded 80% of their heart rate reserve [23]. 

#### 2.2.4. Continuous Test

This procedure was performed on the same treadmill described above at constant speed, using the stage immediately prior to stopping the incremental test [24]. The test was stopped when the participant presented dyspnoea or fatigue ≥ 7 points, a pulse saturometry of <91% and/or exceeded 80% of their heart rate reserve [22]. The information obtained in this test made it possible to determine whether the participant was able to tolerate the load proposed for interval training. Both in the incremental test and the continuous test, the measurements associated with dyspnoea and fatigue were made using a visual analogue scale, according to the Borg scale [19].

## 3. Pulmonary Rehabilitation Program

The exercise sessions were carried out twice a week in person for two months (November 2020 to January 2021). Each face-to-face session was divided into 30 min of aerobic training, 20 min of strength training and 10 min of flexibility training. The sessions were individual, directed and supervised by a physical therapist. In addition, the inspiratory muscle strength training was performed by the patient at home under the indication of the physical therapist. Workloads in aerobic exercise were performed with the results of the previously described incremental and continuous tests.

### 3.1. Face-To-Face Sessions

#### 3.1.1. Aerobic Training

Prior to each session, bronchodilation was performed with 200 mcg of salbutamol, because no patient had saturation problems or was an oxygen user. Oxygen was only used in aerobic training; it was supported with two litres of oxygen through the nose or according to the pulse oximetry of each patient in exercise. Aerobic training was performed on a treadmill. The work strategy used was interval training, where 60% and 80% of the speed and inclination obtained in the incremental test were maintained for two and three minutes, respectively [22]. The criteria for stopping the training session were the same used in the incremental and continuous tests, in addition to the symptoms of inadequacy to exercise such as dizziness, headache and pain. Aerobic workloads were used from the values obtained in the incremental and continuous tests (Table 1).

#### 3.1.2. Strength Training

In the first instance, a warm-up and joint mobility of the whole body were performed, after which lower limb exercises were performed using semi-squats and medium-resistance elastic bands (green; Theraband, Hygenic Co., Akron, OH, USA), and finally, muscle chain exercises were performed bilaterally (biceps, triceps, trapezius, latissimus dorsi, abdominals and hip abductors). An exercise progression sequence was followed, starting with two sets of 10 repetitions, with 20 s of rest between each series, to conclude the intervention with three sets of 25 repetitions and 20 s of rest between each series (Table 1) [25].

#### 3.1.3. Flexibility Training

Each session ended with flexibility exercises that consisted of muscle stretching for each muscle group worked (2 series × 15 s of maintenance), concentrating on the inhalation and exhalation process [26]. If the patient reported joint pain after the training session, cryotherapy was used for 20 min (Table 1) [27].

### 3.2. At Home Sessions

#### Inspiratory Muscle Training

This was performed at home with a threshold valve (Philips Respironics, NJ, USA) IMT (Inspiratory Muscle Trainer), twice a day (morning: between 7:00 a.m. and 12:00 p.m.; afternoon: between 4:00 p.m. and 9:00 p.m.), for each day of the eight-week pulmonary rehabilitation program. The threshold valve was set at 30% of the initial maximum inspiratory pressure (MIP). The established training protocol consisted of three series of 3 min of training with 2 min of rest; breaths should be slow and deep. The evaluation and progression of the training had to be recorded in a daily record guideline, which was evaluated by the physical therapists in charge of each face-to-face session. Training load was readjusted weekly and MIP was reassessed at the end of the program (Table 1) [28].

## 4. Safety

Work was carried out in a 24 m^2^ box with an exhaust fan, under the care standards implemented by the de Hospital El Carmen in Maipú, Santiago, Chile, during the pandemic, where only the kinesiologist and the patient were present. Rotation was every 60 min and all instruments were cleaned with 70% isopropyl alcohol. The kinesiologist used an N95 mask and face shield and the patient used an N95 mask (3M, St. Paul, MN, USA).

## 5. Statistical Analysis

The results are presented as means, standard deviation and 95% confidence intervals. The statistical program used was STATA 16 (StataCorp. Stata Statistical Software, College Station, TX, USA). The normality of data was determined through the Shapiro–Wilk test. For the statistical analysis of HR in the 6 MWT, the Student *t*-test or Wilcoxon test for paired samples was used. For the analysis of HR between training sessions, ANOVA was used for repeated measures. For the analysis of dyspnoea and fatigue data, the Friedman test was used. Correlations were established using the Pearson or Spearman coefficient, depending on the normality of the data. A significance level of *p* < 0.05 was considered.

## 6. Results

### 6.1. Anthropometry and Spirometry

Twenty-two patients were trained, but two did not complete the eight-week intervention due to voluntary withdrawal, so the results of 20 patients (11 men and 9 women) were analysed. The baseline anthropometric and lung function characteristics of the patients evaluated are shown in Table 2. Weight, body mass index (BMI) and FVC significantly increased after the pulmonary rehabilitation program (Table 3).

### 6.2. Heart Rate and 6-Minute Walk Test Performance

RHR significantly decreased from 81.95 ± 9.36 (95% CI: 77.57–86.33) to 73.60 ± 9.82 (95% CI: 69.00–78.20) beats/min (*p* = 0.0008). HRR increased significantly from 12.45 ± 10.22 (95% CI: 7.66–17.23) to 20.55 ± 7.33 beats/min (95% CI: 17.12–23.98) (*p* = 0.005). HR6 min and HR6 min^×^ SBP did not show significant differences after the pulmonary rehabilitation program (*p* > 0.05). Both walking speed and walked distance in the 6 MWT increased significantly from 4.70 ± 1.15 (95% CI: 3.98–5.41) to 5.73 ± 0.99 km/h (95% CI: 5.26–6.19) (*p* < 0.001) and from 451.5 ± 152.2 (95% CI: 380.2–522.7) to 549.3 ± 83.04 (95% CI: 510.4–588.1) metres (*p* < 0.001), respectively (Table 4). 

### 6.3. Control Parameters between Pulmonary Rehabilitation Sessions

**Figure 1:**
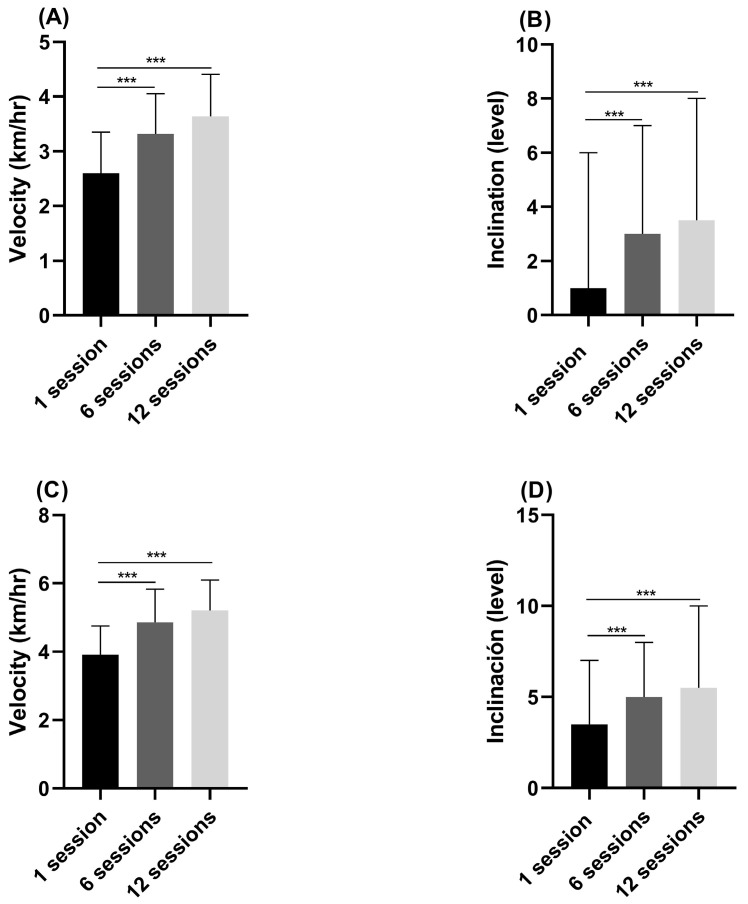
Workloads in pulmonary rehabilitation program sessions. (**A**) Low speed load; (**B**) low tilt load; (**C**) high speed charge; (**D**) high Tilt Load; ***: *p* < 0.001.

**Figure 2:**
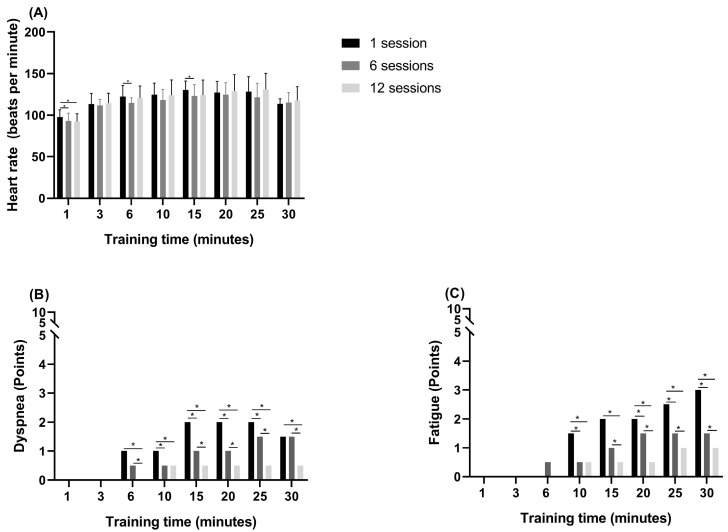
Cardiac response and perceptions in the respiratory rehabilitation program sessions. (**A**) Heart rate; (**B**) dyspnoea; (**C**) fatigue; *: *p* < 0.05.

Between sessions 1 and 6 of the pulmonary rehabilitation program, a significant decrease in HR was observed at minutes 1, 6 and 15. In addition, a significant decrease in HR was observed between sessions 1 and 12 at minute 1. The walking speed in each session increased significantly from session 1 to 6 (*p* = 0.040) and from session 1 to 12 (*p* < 0.05) (Figure 1A,C). Similarly, the incline increased significantly from sessions 1 to 6 (*p* < 0.05) and from sessions 1 to 12 (*p* < 0.05) (Figure 1A,C). The subjective sensation of dyspnoea decreased significantly between sessions 1 and 6 at minutes 6, 10, 15, 20, 25 and 30 and between sessions 1 and 12 at minutes 10, 15, 20, 25 and 30 (Figure 2B). The subjective feeling of fatigue decreased significantly between sessions 1 and 6 at minutes 10, 20, 25 and 30 and between sessions 1 and 12 at minutes 10, 15, 20, 25 and 30 (Figure 2C).

### 6.4. Relationship of the Products of Heart Rate with Speed and Walked Distance in the 6-Min Walk Tests

HR6 min showed significant relationships before and after the pulmonary rehabilitation program. The relationships with speed and walked distance prior to the pulmonary rehabilitation program were r = 0.535, *p* = 0.015 and r = 0.528, *p* = 0.016, respectively. After the pulmonary rehabilitation program, the relationships of HR6 min with speed and walked distance were r = 0.555, *p* = 0.011 and r = 0.613, *p* = 0.011, respectively (Table 5). The HRR with the speed and distance walked before the pulmonary rehabilitation program did not show any significant relationships. However, after pulmonary rehabilitation, HRR had a significant relationship with walked distance (r = 0.461; *p* = 0.040) (Table 5). The HR6 min^×^SBP, both before and after the pulmonary rehabilitation program, showed significant correlations with walking speed (PRE: r = 0.457, *p* = 0.042 and POST: r = 0.538, *p* = 0.014) and distance travelled (PRE: r= 0.528, *p* = 0.016 and POST: r = 0.568, *p* = 0.008) (Table 4).

## 7. Discussion

The aim of this research was to analyse the usefulness of HR during the evaluation and development of a pulmonary rehabilitation program in severe COVID-19 patients who survived an MV stay. The main findings were the significant decrease in HRH and the significant increase in HRR after 8 weeks of an individualised and supervised pulmonary rehabilitation program in patients who were severe COVID-19 survivors of MV. There was also a direct and significant relationship between HR6 min, walking speed and walked distance in the 6 MWT, before and after the rehabilitation process. In addition, a significant decrease in the HR of velocity and slope was observed between sessions 1, 6 and 12, accompanied by a significant decrease in the perception of dyspnoea and fatigue. In this line, the importance of this study is that it makes it clear that the beneficial results obtained here reinforce the concept of personalised training. 

Gruet et al., set out to determine whether maximal HR during the 6 MWT could be used to predict the gas exchange threshold HR during a maximal cardiopulmonary exercise test in patients with cystic fibrosis. Their results showed that there were no significant differences between HR6 max at 6 MWT and gas exchange threshold HR at maximal cardiopulmonary effort. They also observed a direct and high relationship between the maximum HR in the 6 MWT and the gas exchange threshold HR in both patients with cystic fibrosis (r = 0.91; *p* = 0.01) and the control group (r = 0.81; *p* = 0.01) [9]. In addition to this, Pepera et al., showed that patients with chronic heart failure have a shorter step length and walk more slowly than controls during the 6 MWT. Altered gait mechanics can contribute to limited exercise capacity in patients with chronic heart failure [29]. This coincides with the results of the present investigation, which indicate that there was a direct and significant relationship between HR6 min and the speed and walked distance in the 6 MWT. Although the HR6 min did not present significant differences after the pulmonary rehabilitation program (*p* = 0.381), the RHR decreased significantly after the pulmonary rehabilitation, a fact that is considered one of the benefits of physical exercise on the cardiac system [13]. This could have “delivered a greater number of heartbeats” at the time of performing the 6 MWT, a fact that could have resulted in a significant increase in speed and distance obtained in the 6 MWT.

On the other hand, the recovery period also showed a significant relationship with the walked distance in the 6 MWT after the pulmonary rehabilitation program. In this regard, Pepera and Panagiota investigated the effects of habitual smoking on heart rate response and HRR after the step test in athletes. Their results indicate that athletes–smokers had a higher RHR (*p* < 0.05) and lower HRR (*p* < 0.04) in relation to athletes–non-smokers. From these results, they concluded that these changes contribute to the adaptation of cardiovascular function to training requirements [30]. On the other hand, Morita et al., compared the physical activity patterns and functional status of COPD participants with or without late recovery of HR after 6 MWT. Their results indicated that patients with a recovery of less than 12 beats/min in the first minute after the end of the 6 MWT have a significant decrease in the walked distance in the 6 MWT versus patients who have a recovery of over 12 beats/min [20]. The present investigation reported a direct and significant relationship between the walked distance and the recovery in the first minute of recovery after the 6 MWT; that is, the greater the beats/min (≥12) of recovery, the greater the distance covered in the 6 MWT. Although it is not possible to identify the cause of the delay in the recovery of HR, Morita et al., linked it to a sedentary lifestyle and decreased ability to exercise [20]. Although the HRR in this investigation only showed a significant relationship with the distance walked after the pulmonary rehabilitation period, this was accompanied by a significant increase in the HRR after the pulmonary rehabilitation program. This could be due to two facts: (i) the best time per turn in the 6 MWT was used to calculate the walking speed, which would not be representative of the behaviour during the entire 6 MWT, and (ii) the training period included forced walking on an incline treadmill and strength exercises, a fact that improved the performance in metres of the 6 MWT; this overload would have allowed cardiac adaptation, resulting in a rapid return to calm. In this context, the HR6 min would be a good indicator of performance in the 6 MWT, which, when complemented with the HRR, would give a complete view of the behaviour of a subject in the face of maximum exercise.

Although there were no significant differences in HR6 min^×^SBP after the pulmonary rehabilitation program, direct and significant relationships were observed between HR6 min^×^SBP velocity and HR6 min walked distance before and after the pulmonary rehabilitation program. This partially coincides with that reported by Vengatasubramani and Vikram, who investigated the effects of physical training on blood pressure, HR and HR*SBP in COPD participants. The authors studied 30 participants aged between 40 and 55 years; 15 participants were assigned to the experimental group and 15 underwent a pulmonary rehabilitation program consisting of strength exercises. There was a significant difference between the pre- and post-pulmonary rehabilitation values of HR^×^SBP (10,270.67 ± 1379.59 mmHg × beats/min and 8956.80 ± 1162.24 mmHg × beats/min; *p* = 0.028, respectively). This showed that the designed training plan improved cardiovascular fitness in COPD patients [31]. The differences in the results related to HR6 min^×^SBP could be due to the series of secondary disorders of COVID-19 in the cardiovascular system [8]. Due to this background, one of the inclusion criteria for the pulmonary rehabilitation program was an electrocardiogram to rule out heart problems.

Although the HR in training increased during the 30 min of forced walking between sessions 1 and 6, it showed a significant decrease in the three evaluations (session 1, session 6 and session 12), despite there being a significant increase in speed and slope. Senanayake et al., investigated the effects of a 6-week pulmonary rehabilitation program on HR response and metabolic demand in patients with pulmonary fibrosis. After the pulmonary rehabilitation program, there was no significant variability in HR pre-exercise (*p* = 0.14) and during exercise (*p* = 0.12). However, it was observed that the HR significantly increased during the recovery state after the intervention (*p* = 0.036). Furthermore, following the pulmonary rehabilitation program, HR variability increased by 68–75% at rest, exercise and during the recovery period [32]. The results of the present investigation also show an increase in HR in the training sessions, accompanied by a significant increase in speed and incline during the pulmonary rehabilitation program. Despite this, the RHR decreased significantly at the end of the pulmonary rehabilitation program, results that would indicate an adaptation to exercise [8,13]. This difference in the final results, between both investigations, could be due to the different basic pathophysiological conditions, where pulmonary fibrosis results in a deterioration that can be progressive and irreversible. In relation to this, Sima et al., showed that high RHR seems to be an indicator of previous myocardial infarction in patients with chronic lung disease; therefore, careful adjustment of training intensity is recommended under these circumstances [33]. Thus, the effect of training would imply the possibility of achieving an adaptation in patients diagnosed with COVID-19. Therefore, there is a need to describe the behaviour of the cardiac system throughout the rehabilitation process of these patients.

Finally, the results show that dyspnoea exhibits the same behaviour as HR during the training sessions (Figure 2A, B). On the other hand, unlike HR, fatigue and dyspnoea increased steadily throughout the training sessions (Figure 2C). The information available indicates that the perception of dyspnoea and fatigue of the lower limbs increases significantly with a higher workload [34]. In addition, perceptions may increase disproportionately during exercise if gas exchange, cardiac output and/or lower limb musculature fail [35]. Thus, a significant decrease in dyspnoea and fatigue accompanied by a decrease in HR could indicate a better adaptation of ventilatory, cardiac and peripheral musculoskeletal function as patients progress through the sessions. Considering the existence of previous reports that indicate the perception of fatigue in 53% and dyspnoea in 43% of post-COVID-19 patients, training within a period of 60 days is important [36].

Future research that could complement the results obtained here would entail observing the impact on quality of life and have more complex measures such as HR variability or VO_2max_. This research has limitations that need to be discussed. During the critical period of the COVID-19 pandemic, one of the decisions made by the Chilean health authorities was the use of beds in critical units for COVID-19 patients only, so it was not possible to include a control group. This could have generated a potential selection bias. Moreover, the sample of patients studied is low, but it has the strength of being patients who complied with the exercise protocols for 8 weeks and suffered from COVID-19, in addition to being subjected to MV during their illness. The dynamics of health personnel and the redistribution of resources to the closed health system delayed the start of pulmonary rehabilitation, which affected the time of admission to the program. This could have affected the evaluations of lung function and exercise capacity.

## 8. Conclusions

Eight weeks of an individualised and supervised pulmonary rehabilitation program were effective in improving RHR and HRR in COVID-19 patients who survived MV. HR is an easily accessible indicator that could help to monitor the evaluation and development of a pulmonary rehabilitation program in COVID-19 patients surviving MV.

## Figures and Tables

**Table 1:** Description of the pulmonary rehabilitation program.

Face-to-Face Sessions (2×/week)						Home Sessions (Every Day)
Aerobic Training	Strength Training				Flexibility Training	Inspiratory Muscle Training
	Upper-body	Set/ Rep.	Lower-body	Set/ Rep.		
**30 min of walking on a treadmill**.	Bilateral muscle chain exercises of: Biceps	2/10 3/25	Half squats.	2/10 3/25	Two series of 15 seconds for biceps, triceps, trapezius, and latissimus dorsi.	Twice a day: Between 7:00 a.m.–12:00 a.m.: 30 % MIP 3 series of 3 minutes with 2 minutes of rest.
**Interval work**: **2 min at 60% speed and incline obtained in the incremental test**.	Triceps	2/10 3/25	Hip abductors with medium resistance elastic bands	2/10 3/25	Two series of 15 seconds for quadriceps, hamstrings, and triceps surae.	Between 4:00 p.m.–9:00 p.m.: 30 % MIP 3 series of 3 minutes with 2 minutes of rest.
**3 min at 80% speed and incline obtained in the incremental test**	Trapezius	2/10 3/25				
	Latissimus dorsi	2/10 3/25				

Rep: repetitions; MIP: maximum inspiratory pressure.

**Table 2:** Baseline characteristics of the participants.

Variable	Mean ± SD
Age (years)	58 ± 13
Weight (kg)	81.69 ± 15.32
Height (cm)	163.4 ± 8.63
BMI (kg/m^2^)	30.53 ± 4.56
Obesity (*n*/%)	11/55
Diabetes Mellitus (*n*/%)	11/55
Hypertension (*n*/%)	13/65
Smoking habit (*n*/%)	0/0
Length of MV (days)	26.82 ± 14.14
Length of ICU (days)	31.03 ± 15.22
Length of hospitalization (days)	39.94 ± 17.74
Time to enter the program (days)	62.47 ± 30.08

BMI: body mass index; MV: mechanical ventilation; *n*: number; %: percentage; SD: standard deviation; ICU: intensive care unit.

**Table 3:** Spirometric characteristics in COVID-19 participants before and after a pulmonary rehabilitation program.

	PRE	POST	*p* Value
FVC (L)	3.17 ± 0.97	3.30 ± 0.94	0.001 ^w^
FVC pred (%)	86.95 ± 18.94	90.40 ± 16.71	0.001 ^t^
FEV_1_ (L)	2.671 ± 0.84	2.71 ± 0.84	0.558 ^w^
FEV_1_ pred (%)	89.71 ± 25.18	92.95 ± 16.38	0.269 ^w^
FEV_1_/FVC	85.05 ± 5.03	84.25 ± 5.26	0.250 ^t^

PRE: before starting the pulmonary rehabilitation program; POST: after the pulmonary rehabilitation program; FVC: forced vital capacity; FEV_1_: volume that has been exhaled at the end of the first second of forced expiration; L: litres; ^t^: Student *t*-test; ^w^: Wilcoxon test.

**Table 4:** Heart rate, perception and performance of the 6 MWT before and after a pulmonary rehabilitation program.

	PRE		POST		
	Mean ± DS	CI 95%	Mean ± DS	CI 95%	*p* Value
RHR (bpm)	81.95 ± 9.36	(77.57–86.33)	73.60 ± 9.82	(69.00–78.20)	<0.001 ^t^
HR6 min (bpm)	104.6 ± 16.88	(96.65–112.)	107.6 ± 18.50	(98.94–116.3)	0.381 ^t^
HRR (bpm)	12.45 ± 10.22	(7.66–17.23)	20.55 ± 7.33	(17.12–23.98)	0.005 ^t^
HR6 min^×^SBP	15,715 ± 3021	(14,301–17,129)	14,978 ± 3338	(13,416–16,541)	0.218 ^t^
Dyspnoea (points)	3 (1–7)	(2.28–3.71)	3 (0–7)	(1.57–3.52)	0.376 ^w^
Fatigue (points)	3 (0–6)	(2.08–3.91)	2 (0–8)	(1.56–3.73)	0.658 ^w^
Velocity (km/h)	4.70 ± 1.15	(3.98–5.41)	5.73 ± 0.99	(5.26–6.19)	<0.001 ^w^
Walked distance (m)	451.5 ± 152.2	(380.2–522.7)	549.3 ± 83.04	(510.4–588.1)	<0.001 ^w^

The variables are expressed as mean ± standard deviation, dyspnoea and fatigue are expressed as median (minimum–maximum). PRE: before starting the pulmonary rehabilitation program; POST: after the pulmonary rehabilitation program; RHR: resting heart rate; HR6 min: heart rate at minute 6; HRR: heart rate recovery; bpm: beats per minute; SBP: systolic blood pressure; CI: confidence interval; SD: standard deviation; ^t^: Student *t*-test; ^w^: Wilcoxon test.

**Table 5:** Relationship of the products of heart rate and speed and walked distance in the 6 MWT before and after a pulmonary rehabilitation program.

		PRE	POST
		Correlation *p* Value	Correlation *p* Value
HR6 min (bpm)	Velocity (km/h)	0.535 ^p^ 0.015	0.555 ^p^ 0.011
	Walked distance (m)	0.528 ^s^ 0.016	0.613 ^p^ 0.011
HRR (bpm)	Velocity (km/h)	0.256 ^p^ 0.275	0.421 ^p^ 0.064
	Walked distance (m)	0.302 ^p^ 0.194	0.461 ^p^ 0.040
HR6 min*PS	Velocity (km/h)	0.457 ^p^ 0.042	0.538 ^p^ 0.014
	Walked distance (m)	0.528 ^s^ 0.016	0.568 ^p^ 0.008

PRE: before starting the pulmonary rehabilitation program; POST: after the pulmonary rehabilitation program; HR6 min: heart rate at minute 6; HRR: heart rate recovery; bpm: beats per minute; SBP: systolic blood pressure; ^p^: Pearson r coefficient; ^s^: Spearman r coefficient.
